# URO-RESIST: A Real-World Retrospective Study on Multidrug-Resistant Sepsis with Urinary Tract Infection, Clinical Predictors, Inflammatory Biomarkers, and Patient Outcomes

**DOI:** 10.3390/antibiotics14101036

**Published:** 2025-10-16

**Authors:** Livia Moffa, Claudio Tana, Claudio Ucciferri, Samanta Moffa, Jacopo Vecchiet, Katia Falasca

**Affiliations:** 1 Infectious Disease Unit, ASL Lanciano Vasto Chieti and G. D’Annunzio University, 66100 Chieti, Italy; 2 Internal Medicine Unit, Eastern Hospital, ASL Taranto, 74024 Manduria, Italy; 3 Department of Pharmacy, G. D’Annunzio University, 66100 Chieti, Italy

**Keywords:** sepsis, urinary tract infection, multidrug-resistant organisms, C-reactive protein, procalcitonin, carbapenems, empirical antibiotic therapy

## Abstract

Background: Sepsis with Urinary Tract Infection (UTI) is a serious condition with high morbidity and mortality, increasingly complicated by multidrug-resistant (MDR) infections. Prompt diagnosis and appropriate treatment are key to improving outcomes and limiting antimicrobial resistance. The URO-RESIST study aimed to assess the relationship between clinical features, inflammatory biomarkers (C-reactive protein and procalcitonin, CRP and PCT, respectively), microbiological profiles, and outcomes in patients with sepsis with UTI. A secondary aim was to compare microbiological patterns between institutionalized patients and those living at home without known MDR risk factors. Methods: A retrospective cohort study was conducted on 231 patients with sepsis with UTI. admitted to the Infectious Diseases Unit of Chieti Hospital, Italy, between 2014 and 2024 (excluding 2020–2021). Data on clinical, laboratory, and microbiological variables were collected. MDR organisms were defined per European Centre for Disease Prevention and Control (ECDC) criteria. Analyses included non-parametric tests and logistic regression. Results: MDR pathogens were identified in over 40% of cases. Patients from long-term care facilities had higher CRP levels and a higher prevalence of MDR infections. Carbapenems were frequently used, though empirical treatment was sometimes inappropriate. PCT did not differ significantly between MDR and non-MDR groups and had limited prognostic value. CRP and MDR presence were both associated with worse outcomes and longer hospital stays. Functional dependence and dementia were strongly linked to MDR infections. Age correlated with comorbidities but was not an independent predictor of outcome. Conclusions: MDR infections worsen the course of sepsis with UTI. CRP may outperform PCT in assessing severity, especially in institutionalized patients. Functional and cognitive impairments increase vulnerability to MDR pathogens. These findings support the need for rapid diagnostics, targeted antibiotic use, and personalized care strategies.

## 1. Introduction

Sepsis represents a critical clinical syndrome characterized by acute organ dysfunction secondary to a dysregulated host immune response to infection [1]. Specifically, sepsis with urinary tract infection (UTI) originates from infections localized within the urinary tract and is associated with substantial morbidity and mortality. Prompt identification and therapeutic intervention are essential to mitigate complications and reduce hospital length of stay [2]. Reported mortality rates for sepsis with UTI vary between 30% and 40%, which highlights the necessity of early detection and immediate, appropriate management strategies [3]. Although sepsis with UTI can affect patients across all age groups, specific populations, such as individuals with underlying comorbidities such as diabetes mellitus or those with urinary catheters, exhibit increased susceptibility [4]. Sepsis with UTI is classified within the broader category of UTIs, which are stratified based on anatomical localization, clinical severity, and predisposing risk factors [5]. Lower UTIs typically include cystitis and urethritis, while upper UTIs affect renal structures, exemplified by pyelonephritis and renal abscesses [6]. Additional classifications consider the complexity of infection by distinguishing between uncomplicated and complicated cases, recurrent and isolated episodes, symptomatic and asymptomatic presentations, as well as associations with risk conditions such as catheterization, pregnancy, or immunosuppression [7]. The pathogenesis of UTIs commonly begins with colonization of the periurethral area by pathogens derived primarily from fecal microbiota, subsequently ascending the urethra into the bladder [8]. Although the infection may remain localized in the bladder, pathogens can ascend to the renal structures and cause pyelonephritis. In some cases, bacteria invade the bloodstream, which may result in systemic dissemination and progression to sepsis with UTI [4]. Pathogenic microorganisms initiate infection; however, the clinical severity observed in sepsis with UTI primarily arises from the host’s inflammatory and immune responses. Interactions between bacterial components, such as lipopolysaccharides or microbial proteins, and host immune receptors, particularly toll-like receptors, trigger a robust inflammatory response characterized by neutrophil recruitment, cytokine release, endothelial dysfunction, and tissue edema [9]. Despite the growing understanding of sepsis with UTI, a key challenge remains the clinical variability in its presentation and severity, which complicates the assessment of both the condition and the appropriate treatment. The relationship between clinical characteristics, such as comorbidities, and infection severity is not always straightforward. As a result, refining the evaluation of predisposing factors could enhance risk stratification and allow for more targeted, timely interventions.

### Study Objectives

Due to the clinical complexity of sepsis with UTI, the identification of predictive factors for patient outcomes remains essential to optimize management strategies. The primary outcome of the study is to examine the relationship between patients' clinical characteristics, laboratory parameters, and microbial isolates in relation to hospitalization duration and outcomes. As a secondary outcome, the study evaluates differences in microbiological profiles between patients from long-term care facilities and those living at home without risk factors for multidrug-resistant (MDR) pathogens. The analysis defines the clinical profile of patients with sepsis with UTI, establishes correlations between laboratory markers and disease severity, and assesses patient outcomes to support the refinement of treatment strategies. Furthermore, the comparison between different care settings will provide insight into how microbiological profiles and clinical outcomes vary between institutionalized patients and those living at home, contributing to the development of more effective infection control and management strategies.

## 2. Materials and Methods

This single-center cohort study was conducted retrospectively through a review of clinical and microbiological data from patients admitted with sepsis with UTI to the Infectious Diseases Clinic at SS. Annunziata Hospital in Chieti, Italy, within the period between January 2014 and July 2024. Data from the years 2020 and 2021 were excluded from the analysis to ensure sample homogeneity and to minimize bias associated with the elevated number of hospital admissions due to COVID-19. Data were extracted from electronic medical records. These included demographic information (age, sex, comorbidities) and patient origin (home or residential healthcare facilities). Laboratory parameters measured at admission, such as C-reactive protein (CRP), procalcitonin (PCT), and urine analysis results, were recorded. Microbiological findings, with a focus on blood cultures collected at the time of admission, were also analyzed. The adequacy of initial empirical antibiotic therapy was evaluated based on microbiological culture results. Hospitalization duration and clinical outcomes, classified as surviving or dead, were also analyzed. Patients were stratified by origin: home or residential healthcare facilities. The diagnosis of sepsis with UTI was established in accordance with the latest international guidelines [1,10]. These guidelines emphasize early recognition of sepsis and prompt initiation of appropriate antimicrobial therapy, preferably guided by local resistance patterns. They also recommend source control measures, such as removal or replacement of urinary catheters, and careful reassessment of antibiotic therapy once microbiological results become available. The use of biomarkers (e.g., CRP, PCT) is advised as supportive tools for monitoring disease severity and response to therapy [10].

### 2.1. Inclusion Criteria

Patients were eligible for inclusion if they exhibited at least one symptom indicative of UTI, such as dysuria, flank pain, increased urinary frequency, straining during urination, hematuria, or elevated leukocyte esterase on urinalysis. In addition, at least two systemic signs were required, including fever (body temperature > 38 °C) or hypothermia (<36 °C), abnormal white blood cell counts (WBC < 4.0 or >12.0 × 10^9^/L), hypotension (blood pressure < 90/60 mmHg), and positive blood cultures.

### 2.2. Exclusion Criteria

Patients were excluded if they had end-stage renal disease, thyroid carcinoma, no microbiological examinations at admission, negative cultures, or incomplete clinical, demographic, or microbiological data.

### 2.3. Definition of Multi-Drug-Resistant Microorganisms

MDR microorganisms were defined according to the criteria of the European Centre for Disease Prevention and Control (ECDC). According to this definition, a bacterial isolate is considered MDR when it shows acquired non-susceptibility to at least one agent in three or more antimicrobial categories [11].

### 2.4. Statistical Analysis

Statistical analysis was conducted using R software version 4.2.2 (R Foundation for Statistical Computing). Initial descriptive analysis involved the use of exploratory graphical tools, including boxplots, scatterplots, and histograms, to assess trends and distributions. Continuous variables were summarized as means and standard deviations for normally distributed data, or as medians with interquartile ranges (IQRs) for non-normally distributed data. Categorical variables were expressed as percentages. Data distribution was assessed using the Shapiro–Wilk test, which indicated that several variables (e.g., CRP and hospitalization duration) were not normally distributed. As a result, non-parametric tests were applied where appropriate. PCT levels between groups were compared using Welch’s *t*-test for normally distributed data and the Mann–Whitney U test for non-normally distributed data. The association between microbial resistance status (MDR vs. MDS) and hospitalization length was evaluated using the Mann–Whitney U test. CRP levels were similarly compared between patients admitted from home and those from residential facilities using the same test. Logistic regression analysis was applied to assess the relationships between clinical outcomes (complications during hospitalization, discharge without complications, or mortality) and key variables, including PCT, CRP, age, and the presence of MDR microorganisms.

## 3. Ethical Approval

The study protocol was approved by the internal Ethics Committee at the University “G. d’Annunzio” Chieti-Pescara and was conducted in accordance with national regulations and the principles of the Declaration of Helsinki. Informed consent was waived because the study was based exclusively on anonymized, aggregated data collected over a ten-year period, which made it impossible to identify or trace individual patients.

## 4. Results

The study included 231 patients diagnosed with sepsis with UTI between January 2014 and July 2024. This methodological choice enabled a more targeted evaluation of bacterial infections independent of the pandemic. Among the 231 patients, 91 were female (39.0%) and 140 were male (61.0%).

The mean age of the study population was 72.7 years (standard deviation [σ]: 15.12), with a range from 20 to 97 years. The analysis, which included data on the isolation of MDR and multidrug-susceptible (MDS) microorganisms, had MDR microorganisms identified in 95 patients (41.1%), while the remaining 136 patients (58.9%) had MDS microorganisms (Table 1).

An additional analysis, which focused on the origin of the patients, revealed that among the 171 individuals admitted from their own homes, 71 (41.5%) presented with an infection caused by an MDR microorganism.

Of these, only 9 patients (12.68%) had documented risk factors for MDR infections, such as recent urological or surgical interventions (within the previous two weeks), antibiotic use within the past 90 days, hospitalization within the last 90 days, or hemodialysis.

Consequently, 62 patients (36.26%) from the community acquired MDR infections without any identifiable predisposing factors. Among the 60 patients admitted from residential healthcare facilities, 24 (40%) were found to carry MDR microorganisms.

These findings reveal a higher prevalence of MDR microorganisms among patients from residential healthcare facilities (40%) compared to community-dwelling patients without established risk factors (36.26%).

**Figure 1:**
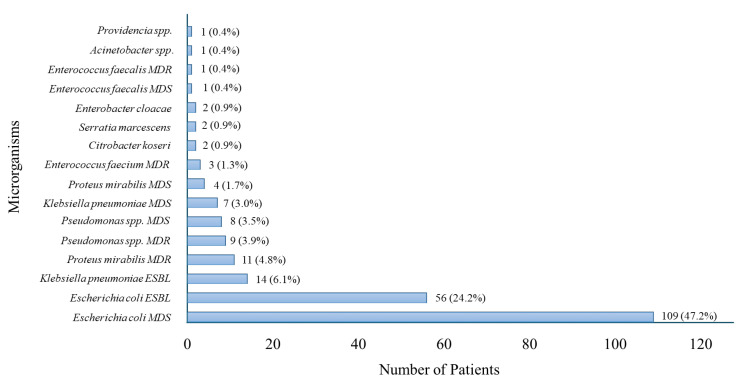
Distribution of Microorganisms Isolated in Patients with Sepsis with UTI. MDR = Multidrug-Resistant; MDS = Multidrug-Susceptible; ESBL = Extended-Spectrum Beta-Lactamase.

The microorganisms isolated from blood cultures among patients with sepsis with UTI are reported in Figure 1.

*Escherichia coli* was the most frequently isolated pathogen, followed by *Klebsiella pneumoniae* and *Enterococcus faecalis*. The chart illustrates the microbiological profile of sepsis with UTI cases, highlighting the predominance of Gram-negative bacteria.

The antibiotics most frequently used were carbapenems (meropenem and ertapenem), administered to 100 out of 231 patients (43.3%). In 69 cases, empirical antibiotic therapy with a carbapenem was later confirmed to be appropriate based on microbiological results. In 26 cases, the initial empirical regimen included one or more agents not supported by microbiological evidence, prompting a subsequent switch to a carbapenem. All of these patients had been admitted from home settings, except for two institutionalized patients. Despite the initial mismatch in empirical therapy, only two patients, both institutionalized, had unfavorable outcomes and died. The remaining 24 patients were discharged at home. Of these, six experienced in-hospital complications, including anemia (3 cases), pulmonary embolism (1 case), atrial fibrillation (1 case), and hospital-acquired delirium (1 case).

In five patients, empirical carbapenem therapy was subsequently deemed inappropriate, as microbiological testing identified MDS microorganisms.

**Figure 2:**
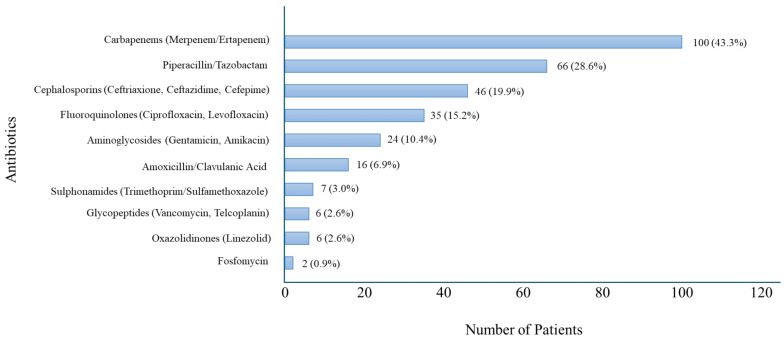
Distribution of Antibiotic Use in Patients with Sepsis with UTI.

Among beta-lactam antibiotics, ureidopenicillin/beta-lactamase inhibitor (piperacillin/tazobactam) was prescribed in 66 patients (28.6%), amoxicillin/clavulanic acid in 16 patients (6.9%), and cephalosporins (ceftriaxone, ceftazidime, cefepime) in 46 patients (19.9%). Fluoroquinolones (ciprofloxacin, levofloxacin) were administered to 35 patients (15.2%), aminoglycosides (gentamicin, amikacin) to 24 patients (10.4%), glycopeptides (vancomycin, teicoplanin) to 6 patients (2.6%), oxazolidinones (linezolid) to 6 patients (2.6%), trimethoprim/sulfamethoxazole to 7 patients (3%), and fosfomycin to 2 patients (0.9%). (Figure 2).

Carbapenems were the most frequently used antibiotics, followed by piperacillin/tazobactam and third- and fourth-generation cephalosporins (ceftriaxone, ceftazidime, and cefepime). The chart reflects empirical treatment choices prior to microbiological confirmation.

Combination therapies were found to be administered to 36 patients (15.6%), primarily including an aminoglycoside (gentamicin or amikacin), a fluoroquinolone (ciprofloxacin or levofloxacin), and an anti-Gram-positive agent (linezolid or vancomycin) in combination with a beta-lactam antibiotic.

Seven patients who received combination therapy developed complications during hospitalization, including anemia, newly diagnosed atrial fibrillation, acute kidney injury, and electrolyte imbalances. One patient died.

### 4.1. Analysis of Procalcitonin (PCT) Levels in MDR vs. MDS Patients

An analysis of PCT levels in patients with MDR infections compared to those with MDS infections revealed several notable observations. On average, PCT concentrations were higher in the MDR group than in the MDS group (25.7 ng/mL vs. 19.4 ng/mL). The median PCT level also appeared elevated in MDR cases (6 ng/mL vs. 4.1 ng/mL) (Table 2). Despite these differences, both groups displayed considerable variability in PCT values, with extreme values affecting both mean and median estimates. Statistical comparisons using Welch’s *t*-test produced *p*-values of approximately 0.13 and above 0.05, respectively. These results did not reach statistical significance, suggesting that the difference in PCT levels between MDR and MDS infections lacked robustness. Additionally, logistic regression analysis assessing the association between PCT levels and the presence of MDR infections yielded no significant results (*p*-value > 0.05), indicating no meaningful correlation. In conclusion, although patients with MDR infections tended to exhibit higher PCT levels, the substantial intra-group variability precluded the identification of a statistically significant difference. Moreover, logistic regression failed to demonstrate the predictive value of PCT levels for distinguishing MDR infections. These findings do not support the use of PCT as a reliable biomarker for differentiating between MDR and MDS infections.

### 4.2. Analysis of Isolated Microorganisms (MDR vs. MDS) in Relation to Hospital Length of Stay

**Figure 3:**
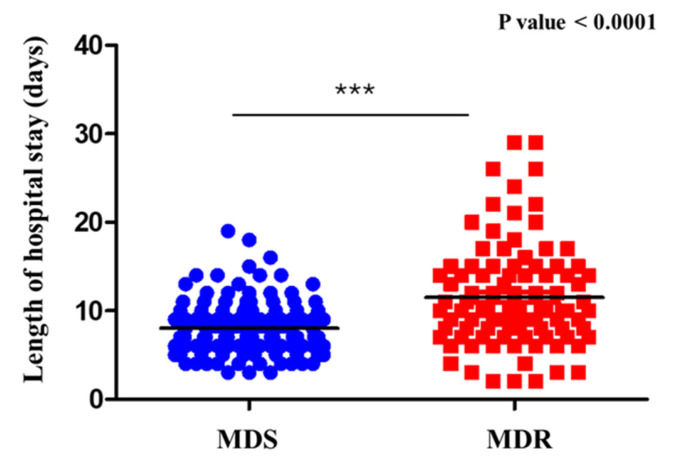
Hospital stay by microrganism type (MDR vs. MDS). Comparison of hospital length of stay between patients with sepsis with UTI caused by MDR and MDS microorganisms. Patients with MDR infections had a significantly longer hospital stay. *** = Statistical significance.

The analysis of hospital length of stay in relation to the type of isolated microorganism (MDR vs. MDS) revealed significant differences. Patients with infections caused by MDS microorganisms had an average hospital stay of 8.51 days (SD = 3.82), with a median of 8 days (range: 2–20). In contrast, patients infected with MDR microorganisms had a longer average hospital stay of 11.33 days (SD = 6.16), with a median of 10 days (range: 2–30). The difference between the two groups was statistically significant (*p* = 5.77 × 10^−5^), as assessed using the non-parametric Mann–Whitney U test. Data distribution had been previously evaluated with the Shapiro–Wilk test, which confirmed that neither group followed a normal distribution (*p* < 0.05). These findings demonstrate a strong association between microorganism resistance profiles and hospitalization duration (Figure 3).

### 4.3. Analysis of CRP Values in Patients from Home Versus Institutional Settings

**Figure 4:**
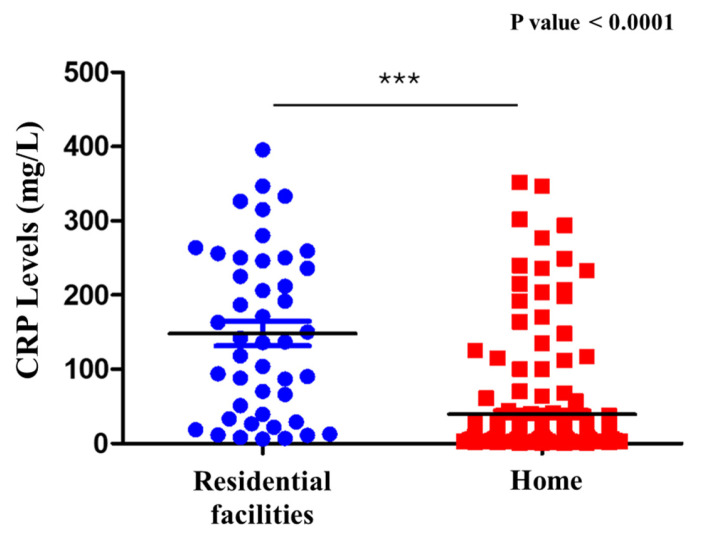
CRP levels in residential facilities and home. Comparison of C-reactive protein (CRP) levels in patients with sepsis with UTI admitted from residential facilities versus those from home. Significantly higher CRP levels were observed in patients from residential facilities. *** = Statistical significance; Blue circles = Residential facilities; Red squares: Home; Black line = Median.

The study population was stratified into two groups based on their origin: 171 were from their own homes, while 60 were admitted from assisted residential facilities (institutions and nursing homes). The results obtained from assessing CRP levels in relation to patient origin, using the Shapiro–Wilk test to evaluate data normality, indicated a non-normal distribution in both groups. Accordingly, the Mann–Whitney U test revealed significantly higher CRP values among institutionalized patients compared to those living at home (*p* < 1.92 × 10^−11^) (Figure 4). To further explore this relationship, Pearson’s correlation coefficient was calculated, revealing a moderate negative correlation between CRP levels and patient origin. This finding suggests that patients residing at home tend to have lower CRP values than those from institutional settings, and that institutionalized patients often present with more severe inflammatory states, as demonstrated by elevated CRP levels. This observation may reflect the increased clinical complexity, more intensive therapeutic requirements, or higher prevalence of comorbid conditions that are commonly found in patients from long-term care facilities.

### 4.4. Analysis of the Relationship Between PCT Values and Length of Hospital Stay

**Figure 5:**
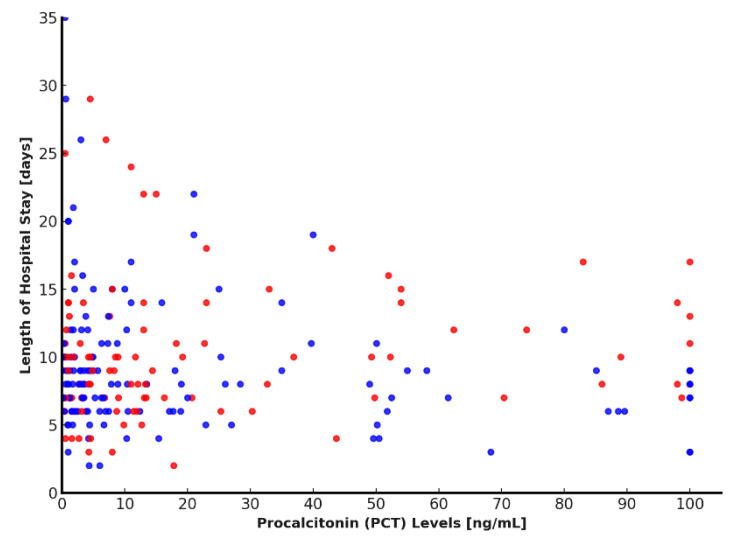
Relationship between PCT levels and length of hospital stay. Scatter plot showing the relationship between PCT levels and length of hospital stay. Higher PCT levels were generally associated with longer hospitalizations. Blue dots represent MDS cases, while red dots indicate MDR cases. PCT: procalcitonin.

The relationship between PCT levels and hospital length of stay was evaluated in the 231 patients included in the study. Linear correlation analysis using Pearson’s coefficient yielded a value of 0.0726, indicating a very weak and non-significant association between PCT concentrations and hospitalization duration. To further explore this relationship, patients were divided into two groups based on the median PCT value (7.59 ng/mL). Comparison of hospital stay duration between the two groups using the non-parametric Mann–Whitney U test showed that patients with higher PCT levels had significantly longer hospitalizations (*p* < 0.0001). This finding was supported by the scatter plot, which demonstrated marked data dispersion, with several extremely high PCT values associated with prolonged hospital stays (Figure 5). Collectively, these results suggest that although the linear relationship between PCT and hospital length of stay appears minimal, patients with higher PCT levels tend to remain hospitalized longer, indicating a potential role for PCT as a marker of clinical severity. Nevertheless, the considerable variability in the data implies the presence of additional influencing clinical factors. Further multivariate analysis is warranted to better delineate the interplay between PCT and other determinants of hospitalization duration.

### 4.5. Analysis of Comorbidities, Age of Patients with Sepsis with UTI, and MDR Microorganisms

**Figure 6:**
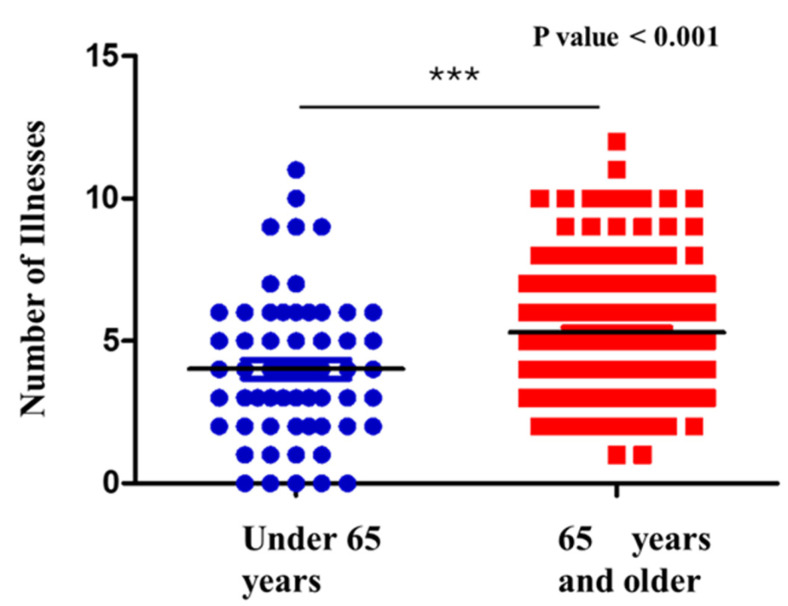
Correlation between number of comorbidities and age. *** = Statistical significance; Blue circles = patients under 65 years; Red squares: patients with 65 years and older; Black line = Median.

Among the 231 patients included in the study, the mean number of comorbidities was 5.2 ± 2.4, with a range from 0 to 17. A statistically significant correlation was observed between age and the number of comorbidities (*p* < 0.001) (Figure 6). This finding indicates that patients with 65 years and older tend to present a greater number of comorbid conditions. The analysis highlights age as a factor associated with the number of comorbidities in the patients examined. This correlation indicates an increase in chronic disease burden with aging. However, it is important to consider other confounding factors not included in this analysis, such as lifestyle, genetics, and access to healthcare. Subsequently, the relationship between patient age and the isolation of MDR and MDS microorganisms was also assessed. No statistically significant association was identified (*p* > 0.05).

Comparison of the number of comorbidities in patients with sepsis with UTI, stratified by age group. Older patients (≥75 years, red) showed a significantly higher burden of comorbidities compared to younger patients (<75 years, blue).

### 4.6. Analysis of the Relationship Between Clinical Outcomes, PCT and CRP Levels, MDR Microorganisms and Age

The prognosis of hospitalized patients with sepsis with UTI may be influenced by multiple clinical factors, such as inflammatory biomarkers, the presence of MDR infections, and advanced age. This analysis investigated the association between these factors and clinical outcomes, which were classified as follows: discharged without complications, discharged with complications during hospitalization, or death. PCT levels did not show a strong correlation with clinical outcomes, suggesting a limited prognostic value in this context. In contrast, CRP levels demonstrated a significant positive correlation (r = 0.488), indicating that higher CRP concentrations were associated with an increased risk of complications or death (*p* < 0.05). The presence of MDR microorganisms exhibited the strongest correlation with unfavorable outcomes (r = 0.707). Age did not differ significantly between outcome groups (*p* > 0.05), suggesting a limited prognostic role in this patient cohort. Multivariate analysis confirmed CRP levels and MDR infections as the most relevant predictors of adverse clinical outcomes, while age did not emerge as a significant factor. Although age is often considered a key prognostic factor, in this dataset, it did not emerge as a statistically significant predictor.

## 5. Discussion

This study included 231 patients diagnosed with sepsis with UTI, analyzing data collected between January 2014 and July 2024. The exclusion of the years 2020 and 2021 was a crucial methodological decision to avoid bias related to the significant impact of the COVID-19 pandemic on both the number and characteristics of hospital admissions as well as on the diffusion of microorganisms [12]. This choice ensured greater sample homogeneity and a more focused analysis of bacterial infections unrelated to the pandemic, providing more reliable and interpretable results. The analyzed sample showed a predominance of male patients, and these findings are consistent with the existing literature, which identifies complicated UTIs as more common in elderly men with comorbidities [13]. Previous studies have highlighted that aging and chronic diseases, such as diabetes and cardiovascular conditions, could increase the risk of sepsis [14,15].

Baseline clinical characteristics revealed that bedridden status and dementia were significantly associated with infections caused by MDR organisms. These findings underscore the heightened vulnerability of functionally dependent and cognitively impaired individuals, who are more likely to have repeated healthcare exposures and prior antibiotic use. This observation aligns with existing literature, which highlights geriatric syndromes and institutionalization as key risk factors for antimicrobial resistance [16].

Another relevant consideration concerns the predominance of elderly male patients in our cohort (mean age 72 years, 60% male). Age-related urological conditions such as benign prostatic hyperplasia, chronic prostatitis, and bladder outlet obstruction are common in this population and can contribute to urinary stasis and incomplete bladder emptying. These mechanisms may predispose to recurrent or persistent UTIs often requiring repeated antibiotic exposure, which in turn may promote colonization or infection by MDR organisms. This pathophysiological background could partially explain the higher frequency and clinical severity of sepsis with UTI observed among older male patients in our study [16].

A notable aspect that emerged from the study was the distribution of isolated microorganisms. Our study showed a significant high prevalence of MDR strains. This result aligns with recent studies reporting increasing MDR rates, including in community-onset and, in some cases, uncomplicated UTIs [17]. Antimicrobial resistance remains one of the most pressing global challenges in infectious diseases, with *Escherichia coli* and *Klebsiella pneumoniae* among the most commonly encountered MDR pathogens [14]. The analysis of antibiotic therapy revealed a predominant use of carbapenems (meropenem and ertapenem), prescribed in nearly half of the patients. This reflects the growing reliance on carbapenems for severe infections, particularly those caused by MDR strains, despite growing evidence supporting carbapenem-sparing approaches [18].

Furthermore, the inappropriate use of carbapenems in some patients with MDS pathogens underscores the importance of timely and accurate microbiological diagnosis. This highlights the urgent need for faster culture sampling and processing protocols, which are often unavailable or delayed in clinical practice, to support more appropriate and targeted antibiotic therapy and help curb the emergence of resistance [19].

Interestingly, among the 26 patients who initially received inappropriate empirical antibiotic therapy, only two experienced an unfavorable outcome (death), both of whom were institutionalized. While this may suggest that adverse outcomes were more closely related to the clinical frailty and complexity of long-term care residents, it does not diminish the importance of timely and appropriate antibiotic selection. In fact, inappropriate empirical therapy still carries risks, such as prolonged illness, complications, and the potential for resistance selection, even in the absence of immediate mortality [19].

The analysis of inflammatory biomarkers revealed some noteworthy patterns. Although PCT levels tended to be higher in patients infected with MDR organisms, this difference did not reach statistical significance. This data is in contrast with other data in the literature showing how PCT levels correlate with death [20,21]. This could be related to the type of district involved and the type of population studied, making the inflammatory process pathogenetically similar for PCT (sepsis predominantly from Gram-negative bacteria). On the other hand, in a study on elderly patients with sepsis, PCT was noted to be accurate in estimating ICU follow-up but was not effective in the prediction of mortality [22]. This suggests that, while PCT is a valuable marker of systemic inflammation, it has limited discriminatory power in identifying MDR infections specifically. Conversely, CRP levels were significantly elevated in patients admitted from long-term care facilities compared to those from home, suggesting a stronger association with underlying clinical severity.

In our cohort, patients with MDR infections experienced a longer duration of hospitalization. This finding is in line with the limited number of studies currently available and supports the emerging evidence that antimicrobial resistance may contribute to prolonged hospital stays. Notably, a study by Madrazo et al. reported similar results in older patients with community-acquired urinary tract infections, highlighting the potential clinical impact of MDR organisms on hospitalization length [23]. The observed trend may be explained by the greater clinical complexity of these patients, who often require second-line antibiotics, prolonged monitoring, and tailored management. However, further high-quality studies are needed to confirm and better quantify this association.

Regarding patient characteristics, advancing age was moderately correlated with a greater number of chronic conditions, highlighting the close relationship between aging and multimorbidity. However, age alone did not emerge as a significant predictor of poor outcomes in this cohort. Instead, the presence of MDR organisms and elevated CRP levels were the most reliable indicators of complications and mortality, reinforcing the critical role of both microbiological profile and inflammatory status in determining clinical prognosis.

### Study Limitations and Strengths

This study presents several limitations that should be considered when interpreting the results. First, its retrospective design relied on previously recorded data, which may be incomplete or subject to documentation bias. Second, the exclusion of data from 2020 to 2021, although intended to ensure greater homogeneity of the sample, may have led to the omission of relevant trends in infection patterns during the COVID-19 pandemic. Third, specific urological data such as prostate-related conditions or urinary tract structural abnormalities, were not collected in this cohort, limiting the ability to explore their potential contribution to infection dynamics. Lastly, the absence of follow-up data precludes the evaluation of long-term outcomes, including delayed therapeutic effects or late-onset complications.

Nonetheless, the study has several notable strengths. It is based on real-world data collected over a ten-year period in routine clinical practice, offering valuable insights into the management of sepsis with UTI in everyday settings. The sample size (231 patients) represents a relatively large cohort for a single-center study and supports the reliability of the findings. In addition, the study population covers a wide age range, allowing for the exploration of age-related clinical patterns. The inclusion of both community-dwelling and institutionalized patients enhances the generalizability of the results across different care settings. Finally, the integration of clinical, microbiological, and inflammatory parameters provides a comprehensive perspective on the prognostic factors associated with sepsis with UTI.

## 6. Conclusions

This study underscores the critical impact of MDR infections in the clinical management of sepsis with UTI, with significant implications for therapeutic choices, hospital stay duration, and patient outcomes. While empirical use of carbapenems proved effective in many cases, the findings highlight the need for more precise and selective antibiotic prescribing to avoid inappropriate use and limit resistance development. Inflammatory biomarkers such as CRP and PCT showed limited ability to distinguish MDR infections; however, elevated CRP levels were associated with greater clinical severity, particularly among institutionalized patients. The separate analyses of hospitalization length and comorbidity burden confirm the clinical complexity of this population and support the need for a multidisciplinary approach in the management of sepsis with UTI. Future research should aim to identify new predictive biomarkers, validate personalized therapeutic strategies, and evaluate interventions to prevent MDR infections.

## Figures and Tables

**Table 1:** Demographic and clinical characteristics of the study population, stratified by infection with multi-drug resistant (MDR) versus multi-drug susceptible (MDS) microorganisms. Data include age, sex, microorganism distribution, and prevalence of major comorbidities. Dementia (*p* = 0.0286) and bedridden status (*p* = 0.0043) were significantly associated with MDR infections.

Age, Year				72.7 ± 15.1
Sex:				
Male				140 (60.6%)
Female				91 (33.4%)
Microorganisms:				
MDR				95 (41.1%)
MDS				136 (58.9%)
Comorbidities:	No. patients (%)	MDR	MDS	*p*-value
Arterial hypertension	121 (52.4%)	48 (50.5%)	73 (53.7%)	0.7355
Dementia	57 (24.7%)	31 (32.6%)	26 (19.1%)	0.0286
Bedridden	42 (18.2%)	26 (27.4%)	16 (11.8%)	0.0043
Renal insufficiency	102 (44.2%)	49 (51.6%)	53 (39.0%)	0.0777
Neoplasms	55 (23.8%)	29 (30.5%)	26 (19.1%)	0.0649
Diabetes mellitus	75 (32.5%)	24 (25.3%)	51 (37.5%)	0.07
COPD	31 (13.4%)	18 (18.9%)	13 (9.6%)	0.0624

**Table 2:** Descriptive statistics of PCT values (ng/mL) in the two groups.

Group	*N*	Mean (±SD)	Median (IQR)	Min–Max (ng/mL)
MDS	136	19.4 (±29.1)	4.1 (1.2–21)	0.07–100
MDR	95	25.7 (±32.0)	6.0 (1.4–30)	0.21–120

MDS = Multidrug-susceptible; MDR = Multidrug-resistant; SD = Standard Deviation; IQR = Interquartile Range.

## Data Availability

The data supporting the findings of this study are available from the corresponding author upon reasonable request.

## References

[1] Singer M., Deutschman C.S., Seymour C.W., Shankar-Hari M., Annane D., Bauer M., Bellomo R., Bernard G.R., Chiche J.D., Coopersmith C.M. (2016). The Third International Consensus Definitions for Sepsis and Septic Shock (Sepsis-3). JAMA.

[2] Wagenlehner F.M., Lichtenstern C., Rolfes C., Mayer K., Uhle F., Weidner W., Weigand M.A. (2013). Diagnosis and management for urosepsis. Int. J. Urol..

[3] Kalra O.P., Raizada A. (2009). Approach to a patient with urosepsis. J. Glob. Infect. Dis..

[4] Flores-Mireles A.L., Walker J.N., Caparon M., Hultgren S.J. (2015). Urinary tract infections: Epidemiology, mechanisms of infection and treatment options. Nat. Rev. Microbiol..

[5] Nicolle L.E. (2013). Urinary tract infection. Crit. Care Clin..

[6] Johnson J.R., Russo T.A. (2018). Acute Pyelonephritis in Adults. N. Engl. J. Med..

[7] Foxman B. (2014). Urinary tract infection syndromes: Occurrence, recurrence, bacteriology, risk factors, and disease burden. Infect. Dis. Clin. N. Am..

[8] Foxman B. (2003). Epidemiology of urinary tract infections: Incidence, morbidity, and economic costs. Dis. Mon..

[9] Van der Poll T., van de Veerdonk F.L., Scicluna B.P., Netea M.G. (2017). The immunopathology of sepsis and potential therapeutic targets. Nat. Rev. Immunol..

[10] Kranz J., Bartoletti R., Bruyère F., Cai T., Geerlings S., Köves B., Schubert S., Pilatz A., Veeratterapillay R., Wagenlehner F.M.E. (2024). European Association of Urology Guidelines on Urological Infections: Summary of the 2024 Guidelines. Eur. Urol..

[11] Magiorakos A.P., Srinivasan A., Carey R.B., Carmeli Y., Falagas M.E., Giske C.G., Harbarth S., Hindler J.F., Kahlmeter G., Olsson-Liljequist B. (2012). Multidrug-resistant, extensively drug-resistant and pandrug-resistant bacteria: An international expert proposal for interim standard definitions for acquired resistance. Clin. Microbiol. Infect..

[12] Falasca K., Vetrugno L., Borrelli P., Di Nicola M., Ucciferri C., Gambi A., Bazydlo M., Taraschi G., Vecchiet J., Maggiore S.M. (2024). Antimicrobial resistance in intensive care patients hospitalized with SEPSIS: A comparison between the COVID-19 pandemic and pre-pandemic era. Front. Med..

[13] Broughton E., Bektas M., Colosia A., Kuper K., Fernandez M.M., Al-Taie A., Kotb R. (2025). A Systematic Literature Review of the Epidemiology of Complicated Urinary Tract Infection. Infect. Dis. Ther..

[14] Rudd K.E., Johnson S.C., Agesa K.M., Shackelford K.A., Tsoi D., Kievlan D.R., Colombara D.V., Ikuta K.S., Kissoon N., Finfer S. (2020). Global, regional, and national sepsis incidence and mortality, 1990–2017: Analysis for the Global Burden of Disease Study. Lancet Lond Engl..

[15] Vallet H., Guidet B., Boumendil A., De Lange D.W., Leaver S., Szczeklik W., Jung C., Sviri S., Beil M., Flaatten H. (2023). The impact of age-related syndromes on ICU process and outcomes in very old patients. Ann. Intensive Care.

[16] Theodorakis N., Feretzakis G., Hitas C., Kreouzi M., Kalantzi S., Spyridaki A., Boufeas I.Z., Sakagianni A., Paxinou E., Verykios V.S. (2024). Antibiotic Resistance in the Elderly: Mechanisms, Risk Factors, and Solutions. Microorganisms.

[17] Ku J.H., Bruxvoort K.J., Salas S.B., Varley C.D., Casey J.A., Raphael E., Robinson S.C., Nachman K.E., Lewin B.J., Contreras R. (2023). Multidrug Resistance of Escherichia coli From Outpatient Uncomplicated Urinary Tract Infections in a Large United States Integrated Healthcare Organization. Open Forum Infect. Dis..

[18] Nguyen C.P., Dan Do T.N., Bruggemann R., Ten Oever J., Kolwijck E., Adang E.M.M., Wertheim H.F.L. (2019). Clinical cure rate and cost-effectiveness of carbapenem-sparing beta-lactams vs. meropenem for Gram-negative infections: A systematic review, meta-analysis, and cost-effectiveness analysis. Int. J. Antimicrob. Agents.

[19] Bassetti M., Kanj S.S., Kiratisin P., Rodrigues C., Van Duin D., Villegas M.V., Yu Y. (2022). Early appropriate diagnostics and treatment of MDR Gram-negative infections. JAC Antimicrob. Resist..

[20] Sun G., Liu W., Zheng Q., Shan Q., Hou H. (2023). Ratio of procalcitonin/Simpson's dominance index predicted the short-term prognosis of patients with severe bacterial pneumonia. Front. Cell Infect. Microbiol..

[21] Ucciferri C., Auricchio A., Cutone C., Di Gasbarro A., Vecchiet J., Falasca K. (2022). Risk Factors Associated with Poor Outcome in Patients with Infective Endocarditis: An Italian Single-Center Experience. Infect. Dis. Rep..

[22] Lee W.J., Woo S.H., Kim D.H., Seol S.H., Park S.K., Choi S.P., Jekarl D.W., Lee S.O. (2016). Are prognostic scores and biomarkers such as procalcitonin the appropriate prognostic precursors for elderly patients with sepsis in the emergency department?. Aging Clin. Exp. Res..

[23] Madrazo M., Esparcia A., López-Cruz I., Alberola J., Piles L., Viana A., Eiros J.M., Artero A. (2021). Clinical impact of multidrug-resistant. BMC Infect. Dis..

